# Formation of multiple ganglion cysts along the flexor tendon after open A1 pulley release for trigger finger: A case report

**DOI:** 10.1097/MD.0000000000029663

**Published:** 2022-07-22

**Authors:** Young Keun Lee

**Affiliations:** Department of Orthopedic Surgery, Research Institute of Clinical Medicine of Jeonbuk National University – Biomedical Research Institute of Jeonbuk National University Hospital, Jeonju, Jeonbuk, Republic of Korea.

**Keywords:** complication, ganglion, flexor tendon, trigger finger

## Abstract

When surgical treatment is indicated for primary trigger finger, open A1 pulley release has traditionally been recommended with generally good results. However, ganglion cysts of the flexor tendon sheath as a complication after an open A1 pulley release were rarely reported. Therefore, the purpose of this study is to report a case of multiple ganglion cysts arising from the flexor tendon sheath in a patient undergoing an open A1 pulley release for trigger finger disorder with a review of the relevant literature.

**Patient concerns and diagnosis:**

A 65-year-old right-handed farmer was referred to our hospital for swelling in the left long finger (LLF). One year before the visit, the patient was diagnosed with trigger finger in the LLF at other hospital and an open A1 pulley release was performed, but the swelling of the finger persisted. The patient had no history of trauma or evidence of systemic disease such as rheumatoid or other inflammatory arthritis. The patient was diagnosed with multiple ganglion cysts of flexor tendon sheath after investigation.

**Intervention and outcomes:**

We successfully excised cystic masses and debrided the partially ruptured flexor digitorum superficialis (FDS) tendon and sutured it using 5/0 prolene. At 12-month follow-up, the patient was completely asymptomatic with excellent range of motion in the distal interphalangeal (DIP) joint (0°–60°) of his LLF, showing no recurrence of ganglion cyst.

**Lessons:**

Trigger finger is a common condition that clinicians encounter frequently. However, this familiarity may lead to inattentive treatment. Nevertheless, through this case, clinicians should devote careful attention when performing open A1 pulley release to prevent partial rupture of the flexor tendon in the A1 pulley. If ganglion cysts occur, we believe that surgical excision can yield good results.

## 1. Introduction

Ganglion may arise in various areas of the hand. However, the flexor tendon sheath of the digit is not a region where ganglion cyst occurs frequently. If it does occur, it is usually a single cyst.^[1]^ In the literature, ganglion cysts associated with stenosing tenosynovitis such as trigger finger are also mostly solitary ganglion cysts.^[2–4]^

Reports of multiple cystic masses in the tendon sheath are very rare. Their occurrence is known to be associated with rheumatic disease.^[5,6]^ To the best of our knowledge, a study by Pannunzio et al^[7]^ is the only report in the literature describing a case of multiple ganglion cysts found in the flexor tendon of 1 digit after surgical treatment of trigger finger not related to rheumatic disease. Trigger finger is a common disorder characterized by the locking or snapping of a digit. Open A1 pulley release is commonly considered as a surgical strategy for trigger finger because it is a simple procedure that yields good results with a very low complication rate.^[8]^ Here we describe a case of multiple ganglion cysts arising from the flexor tendon sheath in a patients undergoing an open A1 pulley release for trigger finger disorder.

### 1.1. Consent

The patient signed informed consent for the publication of this case report and any accompanying images. Ethical approval of this study was waived by the ethics committee of Jeonbuk National University Hospital because it was a case report of fewer than 3 patients (2021-12-043).

## 2. Case presentation

A 65-year-old right-handed farmer was referred to our hospital for swelling in the LLF. He reported no pain in the LLF. However, his fingers were swollen, making it difficult for him to form a clenched fist. If the hand was used repeatedly, the swelling was mitigated and movement became easier. Two months before the visit, aspiration was performed twice using a needle in another hospital. However, swelling in the LLF recurred. One year before the visit, the patient was diagnosed with trigger finger in the LLF at other hospital and an open A1 pulley release was performed, but the swelling of the finger persisted. The patient had no history of trauma or evidence of systemic disease such as rheumatoid or other inflammatory arthritis. His rheumatoid serology was normal.

**Figure 1.**
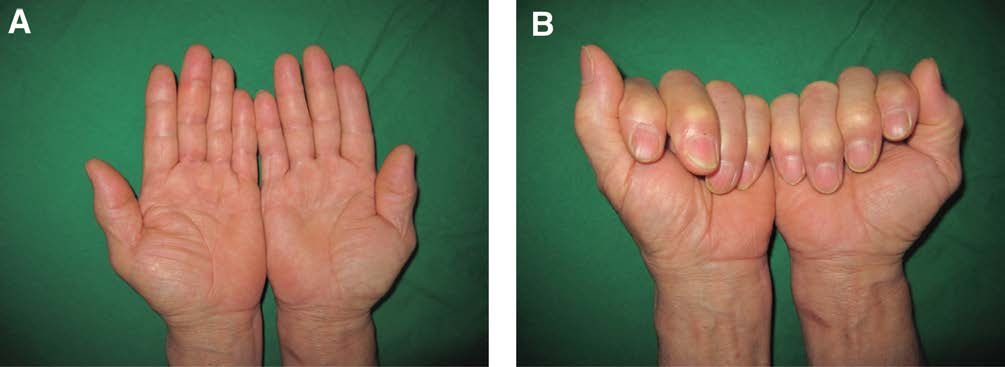
Initial photographs revealing mild swelling in the LLF (A) and approximately 30 degrees flexion in the DIP joint of the LLF (B)

**Figure 2.**
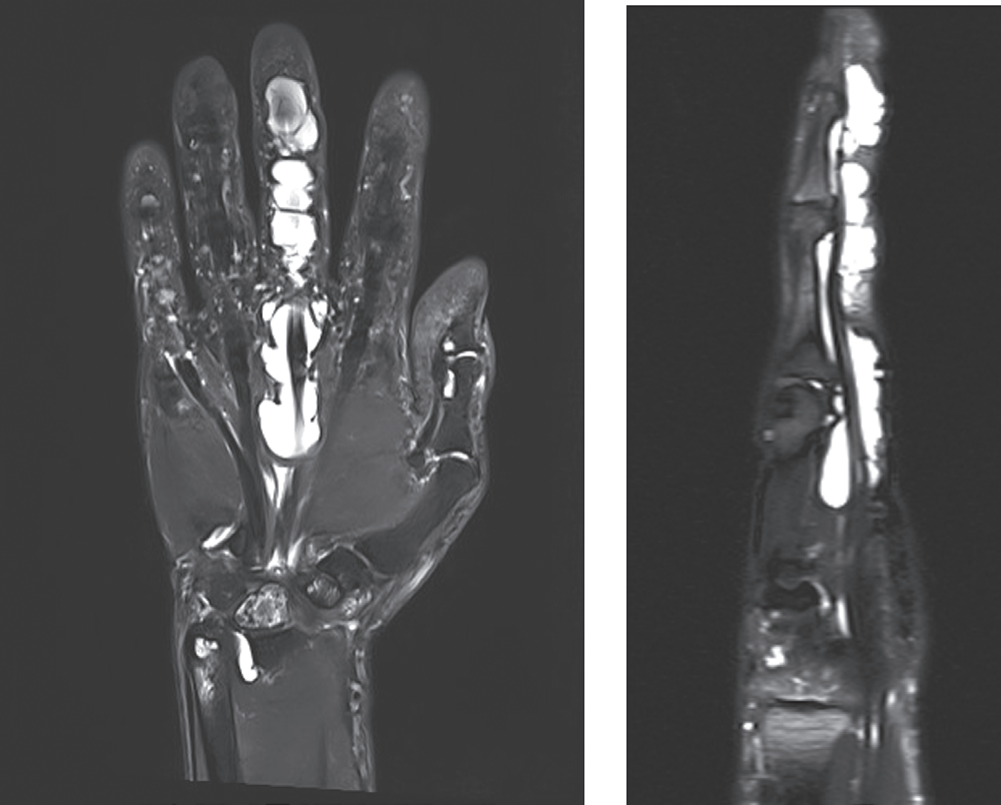
Coronal (A) and sagittal (B) T2-weighted MRI of the left hand revealing severe fluid collection in the flexor sheath, from the metacarpal body to the DIP joint.

Physical examination revealed moderate edema in the LLF and flexion of the DIP joint by approximately 30°. However, there was no tenderness or locking or snapping of the flexor tendon (Fig. 1A, B). Initial plain radiography of the LLF revealed no specific bone or joint abnormalities. Magnetic resonance imaging (MRI) revealed severe fluid collection in the flexor sheath, starting from the metacarpal body to the DIP joint (Fig. 2).

**Figure 3.**
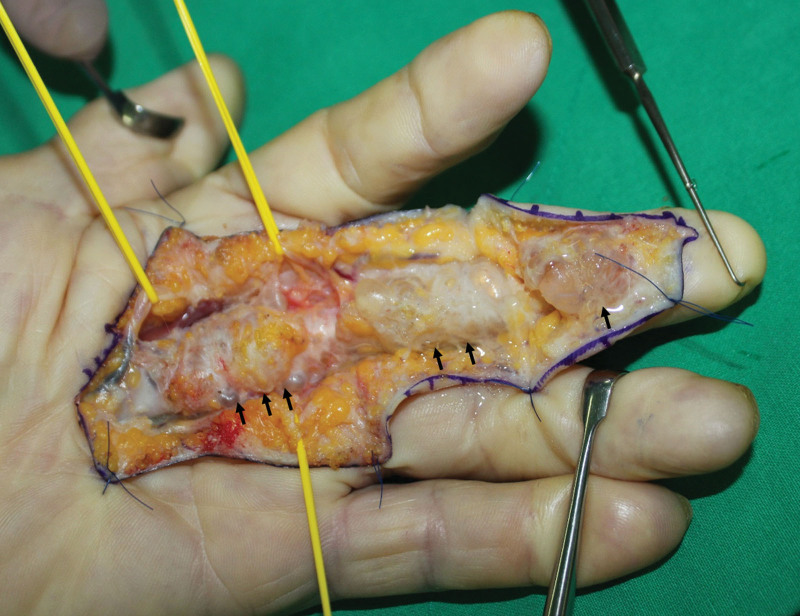
Intraoperative photograph revealing 3 ganglion-like masses with multiple sacs, with the first arising over the A1 pulley, measuring approximately 3 × 1 cm in size, the second located between the A2 and A4 pulley, measuring 2 × 1 cm in size, and the third located immediately distal to the A4 pulley, measuring 1 × 1 cm in size.

**Figure 4.**
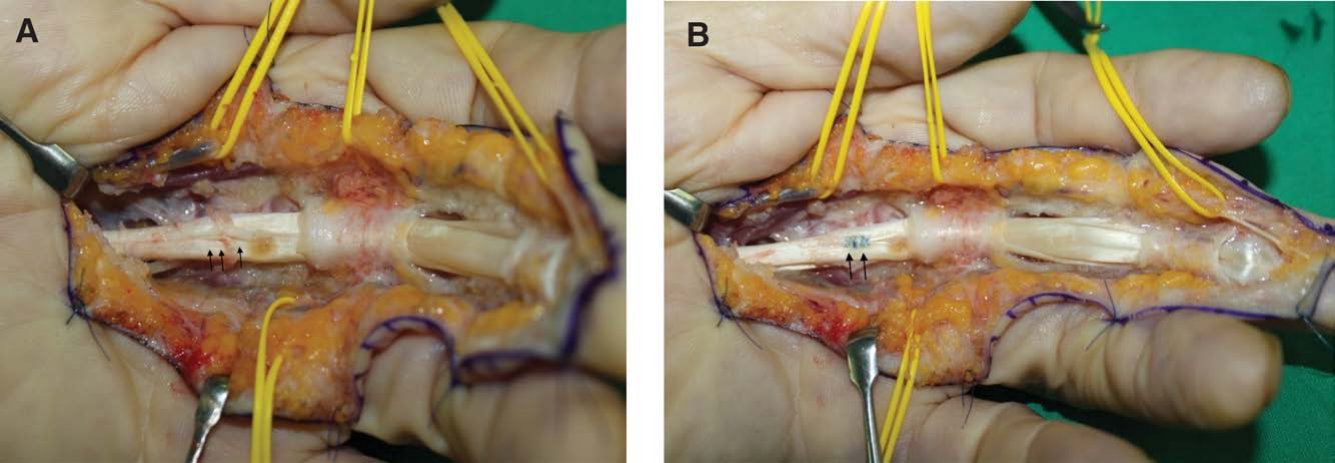
(A) Intraoperative photographs after excision of masses revealing partial rupture and inflammatory change at the site of bifurcation of the FDS tendon. (B) The partial rupture area was debrided and repaired using 5/0 prolene.

**Figure 5.**
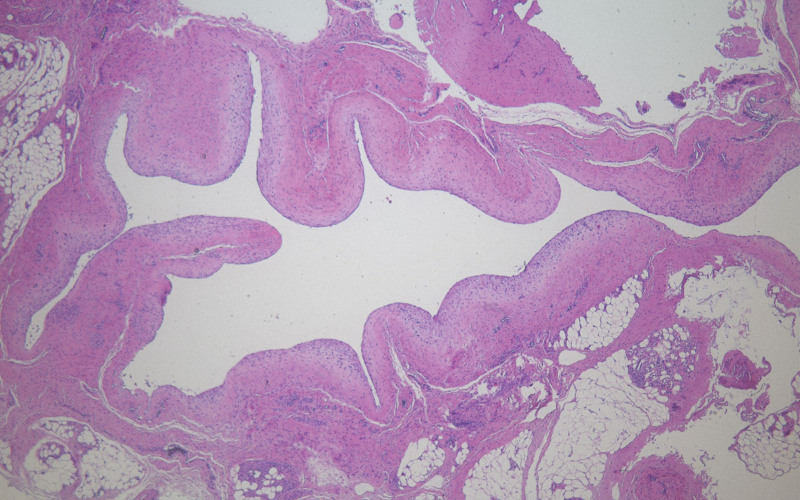
Microscopic appearance of the excised masses revealing fibrotic connective tissue wall without epithelial cell lining (Hematoxylin-eosin, original magnification × 100).

Exploration of the flexor tendon of the LLF was performed using a volar zig-zag incision under regional anesthesia with flexor tenosynovitis impression. During the operation, gross examination revealed 3 ganglion cyst-like masses with multiple sacs. The first 1 was located in the area containing the A1 pulley, measuring approximately 3 × 1 cm. The second 1 was located between A2 and A4 pulleys, measuring approximately 2 × 1 cm. The third 1 was located in an area immediately distal to A4 pulley, measuring approximately 1 × 1 cm (Fig. 3). While preserving A2 and A4 pulleys, all masses were excised, and sent for histological examination. After removing these masses, it was confirmed that the FDS tendon was partially ruptured at the site of bifurcation, and that the site had undergone an inflammatory change. This area was debrided and sutured using 5/0 prolene (Fig. 4). Histopathological examination confirmed that all 3 masses were ganglion cysts (Fig. 5).

After surgery, the LLF was immobilized in an extension-restricted below-elbow plaster splint for 1 week. Flexor dynamic splinting was then applied for an additional 5 weeks. Unrestricted full active motion was permitted at 6 weeks.

**Figure 6.**
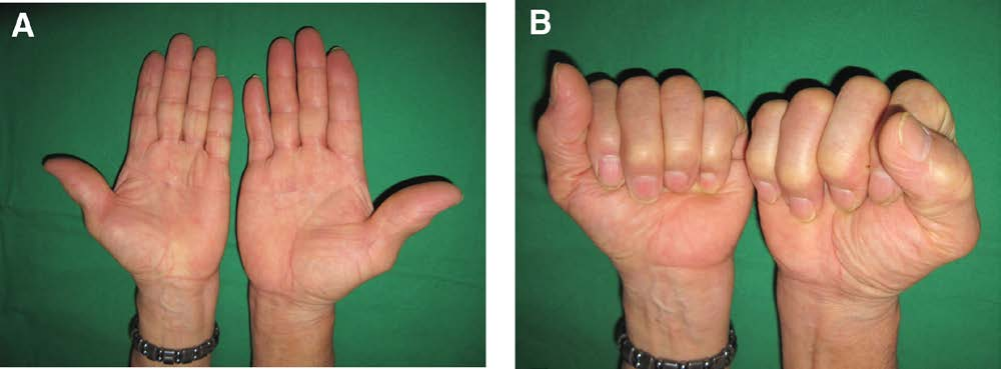
Follow-up photographs obtained at 12- month post operatively revealing excellent appearance of the LLF without recurrence of ganglion cyst.

At 12-month follow-up, the patient was completely asymptomatic with excellent range of motion in the DIP joint (0° to 60°) of his LLF, showing no recurrence of ganglion cyst. Grip strength in the hand was 30 kg (32 kg on the right side). The Quick Disabilities Arm Shoulder and Hand score decreased from 22.7 preoperatively to 6.8 at the final follow-up (Fig. 6).

## 3. Discussion

Ganglion cysts are the most common soft tissue tumors of the hand, with volar and dorsal wrist regions being the most common locations. Involvement of the flexor tendon sheath occurs in approximately 5 to 16% of all hand and wrist ganglion cysts.^[9]^ Regarding the cause of occurrence, explanations including traumatic injury of the flexor tendon or flexor tendon sheath and degenerative changes such as repeated minor trauma have been proposed.^[1]^ However, the exact cause remain unclear.

Ganglion cysts arising from the flexor tendon sheath are usually clinically small (3–8 mm in diameter) with a dome shape. The base is attached to the tendon sheath and unilocular without offshoots from the main cyst. They exhibit little tendency to increase in size with the passage of time. The long finger is the most commonly affected site. It is characterized as painful due to local neural innervation of the cyst.^[1,9,10]^ Histologically, the wall of the ganglion cyst consists of fibrous tissues with a variable number of fibroblasts. There is no membrane lining. At the base of the ganglion, the wall is continuous with the tendon sheath. Microscopic changes in this area are considered to represent changes reflecting degeneration.^[1]^ In our case, it was difficult to determine whether it was clearly histologically different from flexor tendon sheath ganglion cysts. Clinically, because there were 3 masses (not 1) with multiple sacs rather than being unilocular without the presence of pain, it was judged to be a ganglion cyst different from ganglion cysts generally occurring in the flexor tendon sheath.

Trigger finger is believed to be caused by a mismatch between the volume of the flexor tendon sheath and its contents. The diagnosis and treatment of primary trigger finger are well established among most clinicians.^[8]^ When surgical treatment is indicated, open A1 pulley release has traditionally been recommended with generally good results.^[11,12]^ However, a few studies investigating complications of open A1 pulley release have reported complication rates ranging from 11% to 43%.^[11,13–15]^ The majority of reported complications have been minor (including scar pain, tenderness, decreased range of motion, wound redness, or infection that can be treated with oral antibiotics) with recurrence of triggering. However, major complications have also been reported, including bowstringing of the flexor tendon,^[16]^ injury of the digital neurovascular bundle, infections that can be lead to reoperation, synovial fistula, and proximal interphalangeal joint arthrofibrosis.

Thorpe^[13]^ has reported that major complications such as nerve injury and infection can occur following operation performed by junior surgeons. To the best of our knowledge, a study by Pannunzio et al^[7]^ has been the only report of ganglion cyst of the flexor tendon sheath as a complication after an open A1 pulley release. A 57-year-old female received a single steroid injection for trigger finger of the right long finger (RLF). Six months later, triggering recurred and an open A1 pulley release was performed. The patient experienced complete symptom relief for 2 months after the operation. However, at 3 months postoperatively, the patient revisited with complaints of pain and subtle triggering of the volar aspect of the RLF proximal phalanx. Multiple ganglion cysts that occurred in the RLF flexor tendon sheath were diagnosed and treated with complete surgical excision. The authors routinely used an injection volume between 0.5 ml and 1 ml and performed their injection within the sheath between A1 and A2 pulley. They postulated that cysts formed from the pressure exerted within the sheath from the injection and matured to a symptomatic size only after the patient had undergone A1 pulley release. In our opinion, the patient described by Pannunzio et al^[7]^ strictly speaking, might be better regarded as a complication of injection treatment rather than that of the open A1 pulley release. The patient in our case experienced continuous swelling in the finger after undergoing an open A1 pulley release at another hospital. He was not treated even after 2 aspirations at another hospital 2 months before visiting our hospital.

The cause of the formation of multiple ganglion cysts in our patient was speculated to be partial injury at the A1 pulley area of the FDS tendon, which was revealed in surgical findings. Martin and Bensen^[5]^ have reported that synovial cyst can result from a local inflammatory process caused by fluid produced within the bursa or tendon sheath. In other words, synovial fluid might have been continuously generated due to inflammation in the part of the FDS tendon that was partially ruptured as the cause of the formation of the ganglion cyst in this case. As this fluid moved within the tendon sheath, the authors speculated that the cyst was created in the weakened part of the tendon sheath due to pressure, which matured into multiple ganglion cysts.

Recent literature has proposed 2 acceptable treatment options for flexor tendon sheath ganglion cyst: cyst aspiration or surgical excision. However, surgical excision has been advocated due to high recurrence rates reported with aspiration. In addition, surgical excision can avoid the risk of digital nerve injury with a good success rate.^[9,10,17]^ We were able to achieve good results in which the patient was free from recurrence for 1 year after surgical excision.

A limitation of the present study was that inflammatory findings were not presented histologically because a more accurate biopsy was not performed for debridement in the area of the FDS tendon that exhibited inflammation due to partial rupture in surgical findings.

## 4. Conclusions

Trigger finger is a common condition that clinicians encounter frequently. However, this familiarity may lead to inattentive treatment. Nevertheless, through this case, clinicians should devote careful attention when performing open A1 pulley release to prevent partial rupture of the flexor tendon in the A1 pulley. If ganglion cysts occur, we believe that surgical excision can yield good results.

## Acknowledgment

All named authors hereby declare that they have no conflicts of interest to disclose.

## Author contributions

Writing-original draft: Young Keun Lee

Writing-review & editing: Young Keun Lee
